# Aetiology of shame and its association with adolescent depression and anxiety: results from a prospective twin and sibling study

**DOI:** 10.1111/jcpp.13465

**Published:** 2021-06-16

**Authors:** Milica Nikolić, Laurie J. Hannigan, Georgina Krebs, Abram Sterne, Alice M. Gregory, Thalia C. Eley

**Affiliations:** ^1^ University of Amsterdam Amsterdam The Netherlands; ^2^ Lovisenberg Diaconal Hospital Oslo Norway; ^3^ University of Bristol Bristol UK; ^4^ King’s College London London UK; ^5^ National and Specialist OCD and Related Disorders Clinic for Young People South London and Maudsley NHS Foundation Trust London UK; ^6^ Jerusalem Counseling Jerusalem Israel; ^7^ Goldsmiths University of London London UK

**Keywords:** Adolescence, anxiety, depression, twins

## Abstract

**Background:**

Shame is considered a maladaptive self‐conscious emotion that commonly co‐occurs alongside depression and anxiety. Little is known, however, about the aetiology of shame and its associations with depression and anxiety. We estimated, for the first time, genetic and environmental influences on shame and on its associations with depression and anxiety in adolescence.

**Methods:**

The sample was twin and sibling pairs from the Genesis 1219 Study (Time 1, *N* = 2,685; males 42.8%, *M*_age_ = 14.95, *SD* = 1.67, age range: 12–21; Time 2, *N* = 1618; males 39.7%, *M*_age_ = 16.97, *SD* = 1.64, age range: 14–23). Participants completed validated questionnaires to measure shame (at Time 1), depression and anxiety (at Times 1 and 2).

**Results:**

Shame was moderately to strongly associated with concurrent depression and anxiety. Prospectively, shame was significantly associated with an increase in depression, but not anxiety. Genetic analyses revealed that shame was moderately heritable with substantial nonshared environmental influence. The associations between shame and concurrent depression and anxiety were primarily accounted for by overlapping genetic influences. Prospectively, the association between shame and later depression was primarily accounted for by genetic and nonshared environmental influences shared with earlier depression. The unique association between shame and later depression was mostly explained by common nonshared environmental influences.

**Conclusions:**

The findings offer novel evidence regarding aetiology of shame—although moderately heritable, shame in adolescents may also result from nonshared environmental factors. Genetic and nonshared environmental influences contribute to the co‐occurrence of shame with depression and anxiety.

## Introduction

Shame is a self‐conscious emotion that refers to the negative evaluation of the self, typically caused by the violation of social standards (Tangney, 1999). It is characterized by feelings that the entire self is a failure (Lewis, 1992). Individuals who experience shame feel worthless, bad and insignificant and have tendencies to withdraw, hide, or escape (Tangney, 1999). As such, shame is considered a maladaptive self‐conscious emotion that is central to understanding the emotional underpinnings of depression and anxiety (Gilbert, 2000; Kim, Thibodeau, & Jorgensen, 2011). However, little is known about the aetiology of shame and its associations with depression and anxiety. In this study, we estimated, for the first time, the genetic and environmental influences on shame and on its associations with depression and anxiety using twin and sibling data. We investigated this in adolescence—the period when experiencing shame is at its peak and when the rates of depressive and anxiety symptoms increase dramatically (e.g. Ford, Goodman, & Meltzer, 2003).

During adolescence, shame seems to be a particularly salient experience—with increased social concerns and self‐evaluations, temporary disturbances in self‐concept and vulnerability to others’ potentially negative evaluations, adolescents become especially vulnerable to the increased experiences of shame (Reimer, 1996). Existing research suggests that the experience of intense levels of shame is related to increased internalizing symptomatology in adolescence. Experiencing intense shame relates to more depressive symptoms in adolescence, both concurrently and longitudinally (De Rubeis & Hollenstein, 2009; Stuewig & McCloskey, 2005; Tilghman‐Osborne, Cole, Felton, & Ciesla, 2008). A robust relationship between shame and anxiety in adolescence has also been found in childhood and adolescence (Cunha, Matos, Faria, & Zagalo, 2012; Muris, Meesters, Bouwman, & Notermans, 2015). Importantly, there is evidence to indicate that shame mediates the link between psychopathology symptoms and suicidality (e.g. Cunningham et al., 2019; Weingarden, Renshaw, Wilhelm, Tangney, & Dimauro, 2016), suggesting that shame may be an important treatment target to prevent suicidality, which is a major public health concern among adolescents and young adults (World Health Organization, 2014).

Shame may have specific associations with both depression and anxiety. Attesting to this specificity, past studies found that shame is uniquely related to depression after accounting for the symptoms of anxiety (Weingarden et al., 2016) and that shame is uniquely related to anxiety disorder symptoms after accounting for the symptoms of depression (Fergus, Valentiner, McGrath, & Jencius, 2010). From a theoretical point of view, shame may exacerbate the symptoms of depression and anxiety because it involves global and stable negative self‐attributions (Lewis, 1992). First, these negative self‐attributions (e.g. I am a bad person and I will always be bad) may increase risk for developing depressive symptoms because these may contribute to the feelings of helplessness and hopelessness, which are the core of depression (Bennett, Sullivan, & Lewis, 2010). Second, negative self‐attributions may give rise to feelings of inferiority, avoidance behaviours and experiencing the threat of losing social status, all of which are characteristics of anxiety symptomatology (Gilbert & Miles, 2000; Muris et al., 2015). Importantly, the view of the whole self as being negative that is involved in shame (e.g. experiencing oneself as inferior, inadequate) may exacerbate symptoms of both depression and anxiety because it may increase various biases in social information processing (e.g. Bennett et al., 2010; Shahar, Doron, & Szepsenwol, 2015). For example, negative self‐views may give rise to interpretation and attribution bias, negatively interpreting ambiguous social cues and attributing negative events to internal causes (e.g. not being invited to a birthday party of a peer may be interpreted negatively and attributed to internal qualities: ‘I appear boring as I always say stupid things in front of him so he did not want to invite me to the party’). According to the socio‐cognitive models of depression and anxiety in youth (e.g. Hadwin, Garner, & Perez‐Olivas, 2006; Lau & Waters, 2017; Nikolić, 2020; Platt, Waters, Schulte‐Koerne, Engelmann, & Salemink, 2017), these biases in social information processing across childhood and adolescence pose risk for the development of anxiety and depression.

Although it is clear that intense shame is related to both depression and anxiety, and there is some evidence that shame predicts the development of depression from childhood to adolescence (Stuewig & McCloskey, 2005), the mechanisms by which shame influences depression and anxiety symptoms are currently unknown. Moreover, the aetiology of individual differences in shame is not clear. The only family study of shame conducted so far provided evidence of similarity among family members in terms of experiences of shame, which suggests a role of genes and/or the family environment (Johnson, Kim, & Danko, 1989). It is as yet unclear the extent to which genetic versus environmental factors influence the development of shame and its associations with depression and anxiety.

Here, we examined the associations between shame, depression and anxiety in adolescence. First, we examined whether shame is specific to anxiety and depression and whether it contributes to the increases in depression and anxiety. Second, we examined what proportion of variance in the experience of shame is accounted for by genetic and environmental influences. Finally, we tested whether genetic or environmental factors account for the prospective associations between shame and subsequent depression/anxiety symptoms.

## Methods

### Sample

Data from Waves 2 and 3 (from hereon referred to as Time 1 and Time 2) of a longitudinal twin and sibling study, the Genesis 1219 (G1219; McAdams et al., 2013), were used in the present study. Time 1 was chosen because shame was measured only then, and as we aimed to investigate the concurrent and prospective associations with depression and anxiety, Time 2 was also included. The participants in this study were recruited via adults taking part in the GENESiS study (Sham et al., 2000) and from twin registers of the Office of National Statistics. The study was approved by the Research Ethics Review of the Institute of Psychiatry, King’s College, London and Maudsley NHS Trust. Informed consent was obtained from parents of adolescents younger than 16 years old and from participants aged 16 years or older. The first set of analyses, exploring concurrent associations, were conducted using Time 1 data. The sample included 2,685 adolescents who reported on their experience of shame and symptoms of anxiety and depression (males 42.8%, *M*age = 14.95, *SD* = 1.67, age range: 12–21): 616 monozygotic twins, 1,241 dizygotic twins, 185 twins of unknown zygosity and 643 siblings. The second set of analyses, exploring prospective associations, utilized Time 1 and Time 2 data. At Time 2, 1,618 adolescents (males 39.7%, *M*age = 16.97, *SD* = 1.64, age range: 14–23) who reported on their anxiety and depression symptoms were included (the measure of shame was not included at this wave): 373 monozygotic twins, 792 dizygotic twins, 123 twins of unknown zygosity and 330 siblings.

### Measures

#### Shame

An adjusted version of the Experience of Shame Scale (Andrews, Qian, & Valentine, 2002) was used to measure shame proneness at Time 1. The original scale consists of 25 items measuring shame about one’s personal characteristics (e.g. ‘Have you felt ashamed of the sort of person you are?’), shame about one’s behaviours) e.g. ‘Do you feel ashamed when you do something wrong?’) and shame about one’s body (e.g. ‘Have you felt ashamed of your body or any part of it?’). Participants are asked to rate if they have felt what is described in the past year on a four‐point scale (1 = *Not at all*, 4 = *Very much*). The scale was found to be a valid and reliable measure to assess individual differences in experiences of shame. Internal consistency in the original sample was good, α = .90, .87 and .86 for the three subscales, respectively (Andrews et al., 2002). In the adjusted version, 12 items, equally divided across the three shame factors, were selected based on their highest loadings on the corresponding factor from the original analyses (Andrews et al., 2002). Also, all the statements were presented in the first person and in a way that was appropriate for teenagers. The total score was composed and used in the analyses reported here. Internal consistency of the adjusted questionnaire was good in our sample, α = .86.

#### Depression

The short version of the Mood and Feelings questionnaire was used to measure depression in adolescents (MFQ; Angold et al., 1995) at Time 1 and Time 2. The scale consists of 13 highest loading items (e.g. ‘I felt miserable or unhappy’) from the original 33‐item Mood and Feelings Questionnaire (Costello & Angold, 1988). Participants are asked to indicate whether they have experienced symptoms for the past two weeks on a three‐point scale ranging from 0 = *Not true* to 2 = *True*. The scale has been found to reliably predict depressed cases in children and adolescents (Angold et al., 1995) and to have good internal consistency, α = .91 (Sund, Larsson, & Wichstrøm, 2001). In our sample, internal reliability was also good, α = .79/.90 (wave 2/ wave 3). To dichotomize the variable for a subset of our analyses, a cut‐off of > 8 was used to indicate clinically significant symptoms (Thapar & McGuffin, 1998).

#### Anxiety

Spence Children’s Anxiety Scale (SCAS, Spence, 1994) was used to measure anxiety symptoms in adolescents at Time 1 and Time 2. The scale consists of 44 items, of which 38 load on six factors: panic‐agoraphobia (e.g. ‘I suddenly feel as if I can’t breathe when there is no reason for this’), social phobia (e.g. ‘I feel afraid if I have to talk in front of my class’), separation anxiety (e.g. ‘I worry about being away from my parents’), obsessive‐compulsive problems (e.g. ‘I can’t seem to get bad or silly thoughts out of my mind’), physical fears (e.g. ‘I am scared of the dark’) and generalized anxiety (e.g. ‘I worry about things’). Six remaining items relate to positive, filler items to reduce negative response bias. Participants are asked to indicate the frequency of the symptoms occurrence on a four‐point scale from 0 = *Never* to 3 = *Always*. The ratings on 38 anxiety items are summed to provide a total score. The questionnaire was highly reliable, α = .93 in the original sample (Spence, 1994) and α = .87/.94 (Time 1/Time 2) in our sample. To dichotomize the variable for a subset of our analyses, a cut‐off of >33 for boys and >40 for girls was used (Spence, Barrett, & Turner, 2003).

### Data analyses

All variables were log‐transformed to reduce skew (> 1) (see Table 1 for skewness before and after the transformation). We controlled for age and sex in the phenotypic and twin analyses. Age is known to relate to internalizing psychopathology and can therefore lead to twin correlations being artificially inflated relative to sibling correlations. Sex was controlled for because there were significant differences between boys and girls in all study variables (all *p*s < .001).

**Table 1:** Descriptive statistics, full and partial phenotypic correlations between shame, depression and anxiety

Descriptive statistics	*n*	Mean (*SD*)	Skewness (*SE*) before transformation	Skewness (*SE*) after transformation
Shame Time 1	2,623	0.66 (0.41)	0.55 (.05)	0.03 (.05)
Depression Time 1	2,631	7.70 (5.87)	0.95 (.05)	−0.55 (.05)
Anxiety Time 1	2,648	23.85 (13.60)	1.05 (.05)	−0.88 (.05)
Depression Time 2	1,613	6.26 (5.33)	1.14 (.06)	−0.40 (.06)
Anxiety Time 2	1,292	31.24 (12.08)	1.21 (.07)	−0.58 (.07)

Full correlations	Shame Time 1	Depression Time 1	Anxiety Time 1	Depression Time 2
Shame Time 1	—	—	—	—
Depression Time 1	.61 [.55,.66]	—	—	—
Anxiety Time 1	.65 [.60,.70]	.60 [.54,.65]	—	—
Depression Time 2	.41 [.35,.48]	.52 [.46,.59]	.39 [32,.46]	—
Anxiety Time 2	.40 [.34,.47]	.32 [.23,.40]	.55 [.48,.61]	.54 [.47,.60]

Partial correlations	Shame Time 1			
Depression Time 1	.37 [.32,.41]			
Anxiety Time 1	.47 [.43,.51]			
Depression Time 2	.25 [.16,.33]			
Anxiety Time 2	.24 [.16,.32]			

#### Phenotypic analyses

Concurrent and prospective associations between shame and both depression and anxiety were first explored using full and partial correlations. Partial correlations allowed us to investigate the unique associations between shame and depression/anxiety over and above the contribution of anxiety/depression. If shame had a unique association with each type of symptom over and above the other (assessed with partial correlations), each outcome was considered individually from this point onwards in the analyses. As such, linear mixed‐effect models were employed in which depression (or anxiety) at Time 2 was predicted by sex, age, depression (or anxiety) and shame at Time 1 in order to test whether shame prospectively predicts change in depressive/anxiety symptoms, over and above prior symptoms. Mixed‐effects models were chosen to account for clustering of individuals in twin/sibling pairs in our data. Intraclass correlations indicated that 30 and 22% of variance in depression and anxiety, respectively, were explained by individuals being clustered in twin/sibling pairs in our data. Thus, using linear mixed‐effect models to investigate whether shame contributes to the change in anxiety and depression from Time 1 to Time 2 seemed appropriate.

Furthermore, we repeated the analyses using dichotomized depression and anxiety variables which indicated the presence versus the absence of clinically significant symptoms. Thus, this second set of analyses tested whether shame at Time 1 prospectively predicted the odds of having clinically significant depressive/anxiety symptoms at Time 2 after accounting for sex, age and depression/anxiety at Time 1. To account for family clustering within the data, we ran generalized linear mixed‐effects models for the categorical depression/anxiety variables in the same way as we ran the linear mixed‐effects models for the continuous depression and anxiety variables. All phenotypic analyses were performed in IBM SPSS Statistics 25 (IBM Corp., 2017).

#### Genetic analyses

To explore the aetiology of shame and its association with depression and anxiety, multivariate twin analyses were conducted. Twin analyses compare the degree of phenotypic similarity between MZ twins, who share 100% of their genes, with DZ twins and siblings, who share on average 50% of their segregating genes. This design allows the estimation of additive genetic (A), shared environmental (C) and nonshared environmental (E) influences. Assuming an equally similar environment for both MZ and DZ twin pairs/siblings, any increased similarity in MZ twins compared with DZ twins or siblings is attributed to the genetic influences. Within‐pair similarity that is not accounted for by genetic influences is attributed to the effects of the shared environment. Nonshared environmental factors include all nongenetic influences that make the members of the same family different and are estimated from the differences between MZ twins. These factors also include the measurement error (Knopik, Neiderhiser, DeFries, & Plomin, 2016).

For the genetic analyses of Time 1 variables only, we used a multivariate correlated factors solution of the Cholesky decomposition. This model examines the extent to which phenotypic associations between variables are due to correlations between genetic and environmental factors. Thus, with this model, we were first able to estimate what proportion of variance in shame, anxiety and depression was attributable to genetic and environmental factors and then to examine the covariance between these factors as an explanation for the observed phenotypic associations.

For the genetic analyses of the Time 1 and Time 2 variables prospectively, we performed a Cholesky decomposition. This is analogous to the linear mixed‐effect analyses of shame predicting symptom development in anxiety and depression, in that genetic and environmental influences for each variable entered into in the analysis are able to influence all subsequent variables. The ordering of the variables in our Cholesky decompositions was therefore motivated by a combination of our research question and their chronology, and mirrored the approach taken in the linear mixed‐effect analyses detailed above. This allowed us to examine, for example, whether genetic and environmental influences on shame (Time 1) explained variance in later depression (Time 2), after accounting for the shared genetic and environmental influences of earlier depression (Time 1). To maximize power for genetic analyses, we used only continuous (and not categorical) depression and anxiety scores as outcomes.

All genetic models included a scalar to adjust for sex differences in the variances. For each genetic model, we opted not to constrain the full ACE specification (by dropping nonsignificant parameters, as is sometimes done in twin model‐fitting) unless models suffered from convergence problems. Modelling was carried out in OpenMx version 2.12.2 (Neale et al., 2016) running in R version 3.5.1, using full information maximum likelihood estimation to handle missing data.

## Results

### Phenotypic analyses

#### Concurrent associations

Zero‐order and partial correlation coefficients for concurrent associations between shame, depression and anxiety at Time 1 are shown in Table 1 and twin correlations are shown in Table 2. The concurrent associations between shame and depression as well as between shame and anxiety at Time 1 were strong. When accounting for anxiety in the relation between shame and depression at Time 1, as well as when accounting for depression in the relation between shame and anxiety, the correlation decreased to being moderate in strength, but remained significant, suggesting that shame has a unique contribution to depression and a unique contribution to anxiety over and above the influence of the other psychopathological symptoms.

**Table 2:** Twin correlations between shame, depression and anxiety

Twin correlations	Anxiety Time 1	Depression Time 1	Shame Time 1	Anxiety Time 2	Depression Time 2
MZ twin pairs					
Anxiety Time 1	0.60				
Depression Time 1	0.44	0.55			
Shame Time 1	0.43	0.44	0.49		
Anxiety Time 2	0.39	0.33	0.27	0.45	
Depression Time 2	0.30	0.42	0.36	0.22	0.46
DZ twin pairs					
Anxiety Time 1	0.26				
Depression Time 1	0.21	0.27			
Shame Time 1	0.21	0.21	0.22		
Anxiety Time 2	0.18	0.12	0.15	0.11	
Depression Time 2	0.13	0.14	0.13	0.11	0.19

The results are based on the sample of a randomly chosen twin/sibling from the pair.

#### Prospective associations

Zero‐order and partial correlation coefficients for prospective associations between shame at Time 1 and depression and anxiety at Time 2 are shown in Table 1, and twin correlations are shown in Table 2. The prospective associations between shame at Time 1 and depression at Time 2 as well as between shame at Time 1 and anxiety at Time 2 were of moderate strength. When accounting for anxiety at Time 2 in the prospective association between shame at Time 1 and depression at Time 2, the correlation decreased to being low in strength, but remained significant. The same result occurred for the prospective association between shame at Time 1 and anxiety at Time 2, when controlling for depression at Time 1.

To investigate whether shame at Time 1 significantly predicted change in depression from Time 1 to Time 2 after accounting for the influence of sex and age, we ran a linear mixed‐effect model in which we included a random intercept for twin/sibling pair identity. Depression at Time 2 was the outcome, and sex, age, depression at Time 1 and shame at Time 1 were predictors. With the increasing levels of shame at Time 1, the levels of depression at Time 2 also increased after accounting for the influence of sex, age and earlier depression, *F*(1,1554) = 52.45, *p* < .001, and this effect was of medium size (Table 3).

**Table 3:** Parameter estimates for linear mixed‐effect models of depression, anxiety and shame predicting later (a) depression, (b) anxiety

Model	Parameter	β	*SE*	95% CI	*p*
Depression Time 2	Age	−.04	.01	[−.06, −.01]	.008
	Sex	−.24	.04	[−.33, −.16]	<.001
	Depression Time 1	.35	.03	[.30,.41]	<.001
	Shame Time 1	.20	.03	[.15,.26]	<.001
Anxiety Time 2	Age	−.02	.01	[−.05,.01]	.122
	Sex	−.27	.05	[−.37,.−.17]	<.001
	Anxiety Time 1	.53	.04	[.46,.60]	<.001
	Shame Time 1	.07	.03	[−01,.14]	.026

SE, standard error.

With the increasing levels of shame at Time 1, the levels of anxiety at Time 2 also significantly increased after accounting for the influence of sex, age and earlier anxiety, *F*(1,1243) = 4.94, *p* = .026; however, this effect was small in size (Table 3).

We repeated these analyses using a binary depression/anxiety variable at Time 2 (presence or absence of clinically significant symptoms). In the depression model, shame at Time 1 significantly predicted the odds of having clinically significant symptoms of depression at Time 2 over and above the prediction of sex, age and previous levels of depression, *F*(1,1555) = 28.57, *p* < .001, coefficient=0.45, *SE*=.08, 95%*CI* [0.29, 0.62] *p* < .001. In contrast, in the anxiety model, shame did not significantly predict the odds of having clinically significant anxiety symptoms at Time 2 over and above the prediction of sex, age and previous levels of anxiety, *F*(1,1240) = 1.49, *p* = .223, coefficient = 0.12, *SE* = .10, 95%*CI* [−0.08, 0.32] *p* = .223.

### Genetic analyses

#### Concurrent associations

**Figure 1:**
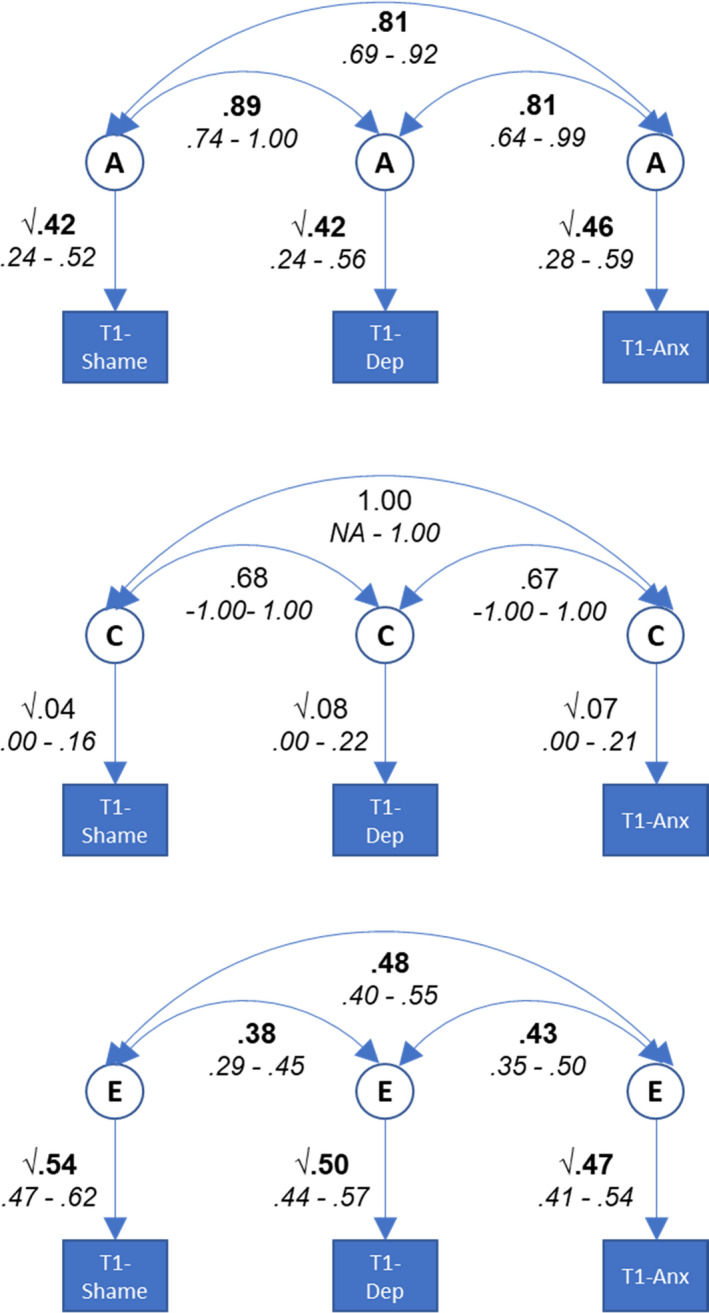
Results from the correlated factor model of shame, depression and anxiety at Time 1 (T1). Note. A genetic effects, C shared environmental effects, E nonshared environmental effects. Significant values in bold face

The correlated factors solution of a trivariate Cholesky decomposition was used to estimate the contributions of genetic and environmental factors to the associations between shame, depression and anxiety at Time 1 (−2LL = 17,442.38, AIC = 2,964.38). The parameter estimates and 95% confidence intervals from this model are shown in Figure 1. Shame was moderately heritable with substantial nonshared environmental influence. Shared environmental influence was not significant. The aetiology of depression and anxiety have been reported previously and were similar to those for shame (for depression see Lau & Eley, 2006; for anxiety see Zavos, Rijsdijk, Gregory, & Eley, 2010). Figure 1 also shows the genetic and environmental correlations among the variables. Genetic influences on shame were highly correlated with those on both depression and anxiety, accounting for 61% [0.38, 0.79] and 55% [0.32, 0.71] of these phenotypic associations, respectively. Nonshared environmental factors influencing variability in shame were also moderately correlated with those influencing both depression and anxiety, and accounted for 32% [0.24, 0.42] and 37% [0.29, 0.46] of these phenotypic associations, respectively. Shared environmental factors accounted for the remainder of the phenotypic associations and were small and nonsignificant for all associations, meaning that irrespective of the estimated correlations between them, their contribution to the phenotypic associations between the variables is negligible. In sum, associations between shame and both depression and anxiety were primarily accounted for by overlapping genetic influences.

#### Prospective associations

**Figure 2:**
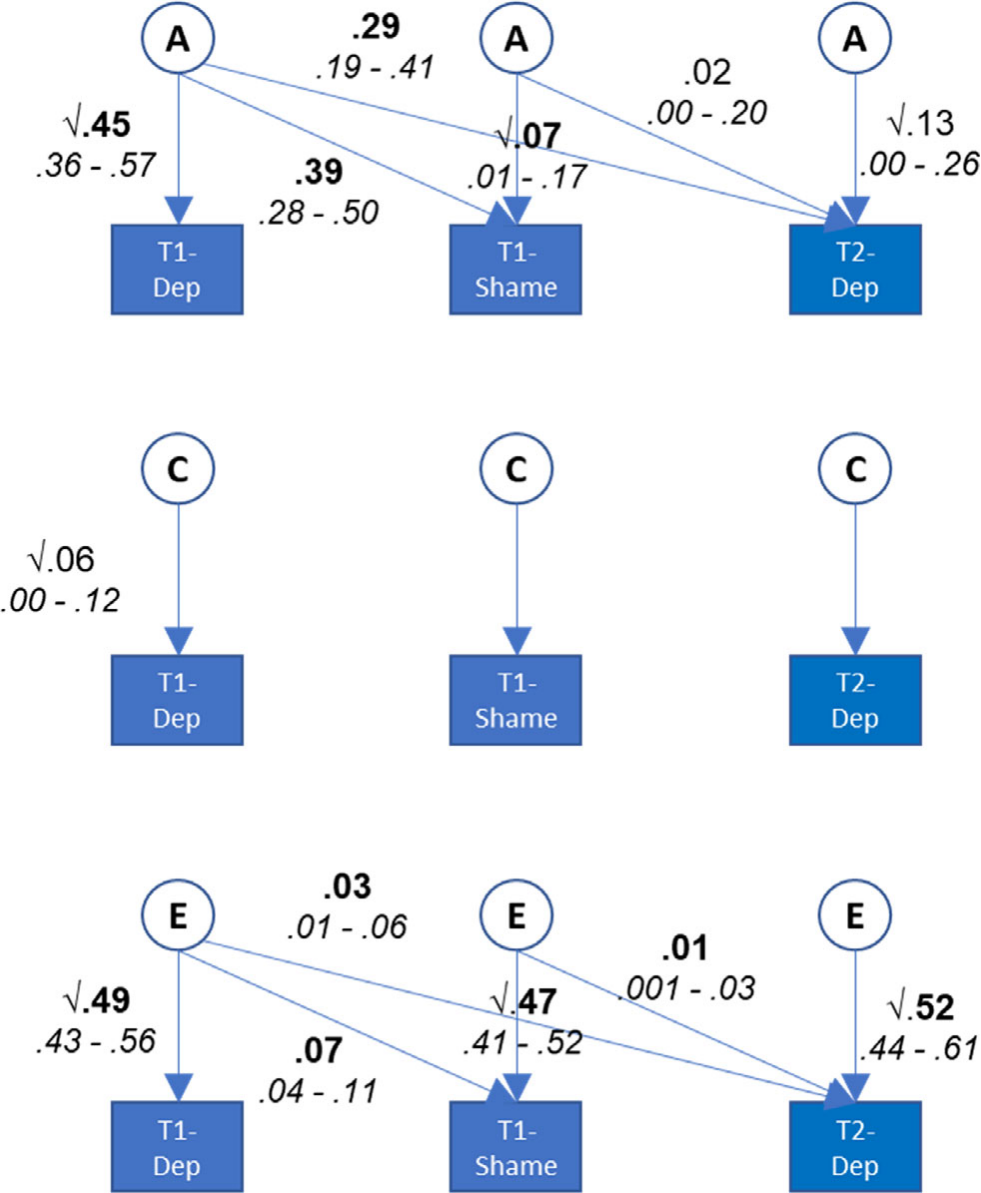
Results from Cholesky decomposition of prospective associations between shame and later depression, accounting for earlier depression. Note. A genetic effects, C shared environmental effects, E nonshared environmental effects. Significant values in bold face. C paths influencing shame at Time 1 and depression at Time 2 (which were originally estimated < .05) were fixed to zero

A trivariate Cholesky decomposition was then used to estimate the aetiology of prospective associations between shame and later depression (−2LL = 16,064.18, AIC = 3,500.18). We did not perform equivalent genetic analyses for prospective associations between shame and later anxiety, because the results of the phenotypic analyses indicated that shame only marginally influenced the change in anxiety levels and did not predict the odds of having clinically significant anxiety symptoms at Time 2 after accounting for the effects of sex, age and earlier anxiety. The parameter estimates and 95% confidence intervals from the model with depression are shown in Figure 2. We constrained to zero all shared environmental influences that were initially estimated at or close to zero (<.03) to help us decompose the unique association between shame at Time 1 and depression at Time 2 from the phenotypic analyses, for which we were underpowered in the full genetic model. This meant that our final model had shared environmental influences on depression at Time 1 only. This simpler model did not result in a poorer fit to the data compared with the full model, ∆χ² (5) = 1.91, *p* = .860. For comparison, the full model is presented in Figure S1. The association between shame and later depression was mostly accounted for by the influence of aetiological factors involved in depression at Time 1. The phenotypic unique association between shame at Time 1 and Depression at Time 2 (after accounting for earlier depression at Time 1) was primarily accounted for by nonshared environmental influences (77%), although this influence was nonsignificant.

## Discussion

The aim of this study was to investigate the aetiology of shame and its associations with depression and anxiety using twin and sibling data. Phenotypic results showed that shame is uniquely associated with both depression and anxiety in adolescence. Furthermore, shame in adolescence predicted an increase in depressive symptoms over time. Shame had a small association with change in the levels of anxiety, but not with subsequent clinically significant symptoms of anxiety, so prospective genetic analyses focused on the association with depression. Twin analyses revealed that genetic and nonshared environmental effects both explained a significant proportion of the variance in shame. Concurrent associations between shame and both depression and anxiety were primarily explained by overlapping genetic factors. Prospectively, although we found a unique phenotypic association between shame and later depression, there were no significant influences on shame and later depression, beyond those shared with earlier depression. However, it should be noted that, although nonsignificant, common nonenvironmental influences accounted for the most of this unique association.

### Phenotypic associations between shame, depression and anxiety

Shame was moderately to strongly associated with both depression and anxiety symptoms. Moreover, shame was uniquely associated with both depression and anxiety symptoms indicating that it may be an independent factor in both types of psychopathology. This is in line with previous studies that reported that shame is uniquely related to depression after accounting for anxiety symptoms (Weingarden et al., 2016) and to anxiety after accounting for depressive symptoms (Fergus et al., 2010). Finally, our results showed that greater levels of shame in early adolescence predicted worsening of depression symptoms by late adolescence, as well as the presence of clinically significant depression symptoms in late adolescence. In contrast, shame in early adolescence had only a marginal association with change in anxiety over time and did not predict the presence of clinically significant anxiety symptoms in late adolescence. The association with depression was expected and is in line with previous research that found that shame prospectively predicts an increase in depressive symptoms in adolescence (Stuewig & McCloskey, 2005). This unique contribution of shame to the increase in depression is especially relevant for clinical practice—it suggests that shame in youth may be a marker for the emergence of later depression. Although further research is needed to establish the mechanisms underpinning the prospective association between shame and depression, our findings raise the possibility that targeting shame in early adolescence may help prevent the development of depression symptoms. In contrast, our results suggest that this is not the case for overall anxiety symptomatology, as we found little evidence that shame in early adolescence independently predicts worsening in anxiety symptoms over time.

### Genetic and environmental influences on shame and its associations with depression and anxiety

This study was the first to examine shame and its associations with depression and anxiety from an aetiological perspective. The results of the twin modelling highlighted moderate significant genetic influence and substantial nonshared environmental influence on shame. The heritability of shame (42%) was similar to that found for related feelings of guilt and hopelessness in a study with adults (Jang, Livesley, Taylor, Stein, & Moon, 2004). Interestingly, the influence of the nonshared environment on shame was substantial, suggesting the importance of the environment alongside genetic factors in the development of shame in adolescents. Nonshared environmental influences reflect child‐specific experiences that can range from specific interactions with parents to the specific influences outside the family, such as stressful life events or peer relations. For example, negative parenting whereby the parent uses verbal disapproval, hostility or physical abuse may result in higher levels of shame experiences in youth (Muris & Meesters, 2014). Considering factors outside the family, shame, especially in adolescence, may result from the low social rank in the peer group because of, for example, unattractive physical appearance, peer victimization or poor school performance (Gilbert, 2000). Finally, shame may result from traumatic life events, such as sexual abuse (Stuewig & McCloskey, 2005). Shared environmental factors seemed not to have an important influence on shame. Similar findings have been reported for other phenotypes associated with depression, such as guilt and hopelessness (Jang et al., 2004) as well as for depression and anxiety symptoms in general (Gregory & Eley, 2007; Rice, Harold, & Thapar, 2002).

The concurrent associations between shame and depression as well as shame and anxiety were explained largely by overlapping genetic factors. Nonshared environmental factors were also significant influences in the associations between shame and both depression and anxiety. The same environmental factors that influence shame may, thus, influence depression and/or anxiety. This is in keeping with findings suggesting that key aspects of the environment, such as parental abuse/neglect or victimization by peers, show quite broad associations with internalizing symptomatology (Bennett et al., 2010).

Although the phenotypic analysis showed that shame adds to the prediction of later depression over and above earlier depression, genetic analyses did not find any significant association between genetic and environmental influences on shame and later depression after accounting for earlier depression. The aetiological influences on earlier depression accounted for most of the association between shame and later depression. This is likely due to the limited power to decompose small‐to‐medium phenotypic associations in our genetic analyses. Nevertheless, it should be noted that the unique phenotypic association between shame and later depression was mostly accounted for by nonshared environmental influences, although this effect was small. This means that the nonshared environmental factors that influence shame may worsen the depression symptoms in adolescence in addition to the shared genetic and environmental influences with earlier depression. It could also mean that shame has a causal influence on depression via nonshared environmental factors. For example, adolescents who experience high levels of shame may start to withdraw from social activities and get fewer rewarding experiences from their environment or get rejected by it. This may place them at risk for depression. If this were the case, it would be helpful to include shame as an intervention target in prevention programmes for depression. This is particularly important given that shame mediates the risk for suicidality and that it can be effectively reduced with psychological interventions, such as compassionate mind training (e.g. Gilbert & Procter, 2006).

### Limitations

Our findings should be interpreted in view of some limitations. First, we used questionnaire data to measure shame, depression and anxiety. Therefore, the results hold for self‐reported shame, depression and anxiety symptoms, and they cannot be generalized to disorders. Results should be replicated in clinical samples using clinical diagnoses. Also, by relying on self‐reported data, the correlations could be inflated due to the shared method variance and the common genetic factor of shame, depression and anxiety may partly reflect the shared method variance. Second, we did not measure shame at both time points, which precluded conclusions about the reciprocal longitudinal effects of shame on depression and anxiety and vice versa. Nevertheless, we measured anxiety and depression at both time points which allowed us to examine whether shame influences the development of depression/anxiety across adolescence, which was the main question of this study. Future studies should explore whether shame, depression and anxiety mutually influence each other over time to further build on our findings. Third, although the sample size was relatively large and we had enough power to detect medium‐sized effects in our phenotypic analyses and most of our genetic analyses, we did not have enough power to decompose the small but significant unique prospective association between earlier shame and later depressive symptoms in the twin models while estimating all genetic and environmental variance and covariance parameters for three variables. To account for this, we constrained some of the shared environmental paths to zero. Making such constraints in twin models allows remaining parameters to be estimated more precisely, at the cost of inflating genetic path estimates (to the extent that constrained C paths were estimated at > 0 in the full model). We opted to accept this cost because, in this case, the main covariance parameters that we needed to interpret in the constrained model (A/E covariance between shame at Time 1 and depression at Time 2) should not have been inflated since shared environmental covariance between these variables in the full model was estimated at exactly zero (see Supporting Information). In addition, as we were not able to examine specific genetic variants or specific environmental influences on shame and its association with depression and anxiety in our twin study, other designs with measures of specific genetic variants or specific environmental influences are needed to investigate which specific genetic and environmental factors contribute to shame and its associations with depression and anxiety. Finally, the limitations related to twin designs in general, such as whether we can generalize the findings on twins to the general population, could also influence our study.

## Conclusions

Genetic and nonshared environmental factors were most important in explaining the aetiology of shame in adolescents. Thus, although moderately heritable, shame may be socialized through child‐specific experiences such as traumatic and stressful life events in and outside the family. Considering that shame is strongly related to depression and anxiety due to overlapping genetic and nonshared environmental influences, and that shame uniquely contributes to the increase in depression in adolescence, efforts to prevent and treat feelings of shame in preadolescence may prove useful in curbing adolescents’ depression.

## Supporting information


**Figure S1**. Results from Cholesky decomposition of prospective associations between shame and later depression, accounting for earlier depression.
