# Supplementary material for: Aetiology of shame and its association with adolescent depression and anxiety: results from a prospective twin and sibling study
Source: J Child Psychol Psychiatry. 2021 Jun 16;63(1):99–108. doi: 10.1111/jcpp.13465 (PMC9292396; doi:10.1111/jcpp.13465)
Supplement: Supplementary file 1 — Figure S1. Results from Cholesky decomposition of prospective associations between shame and later depression, accounting for earlier depression. [file JCPP-63-99-s001.docx]

**Supporting Information**

**
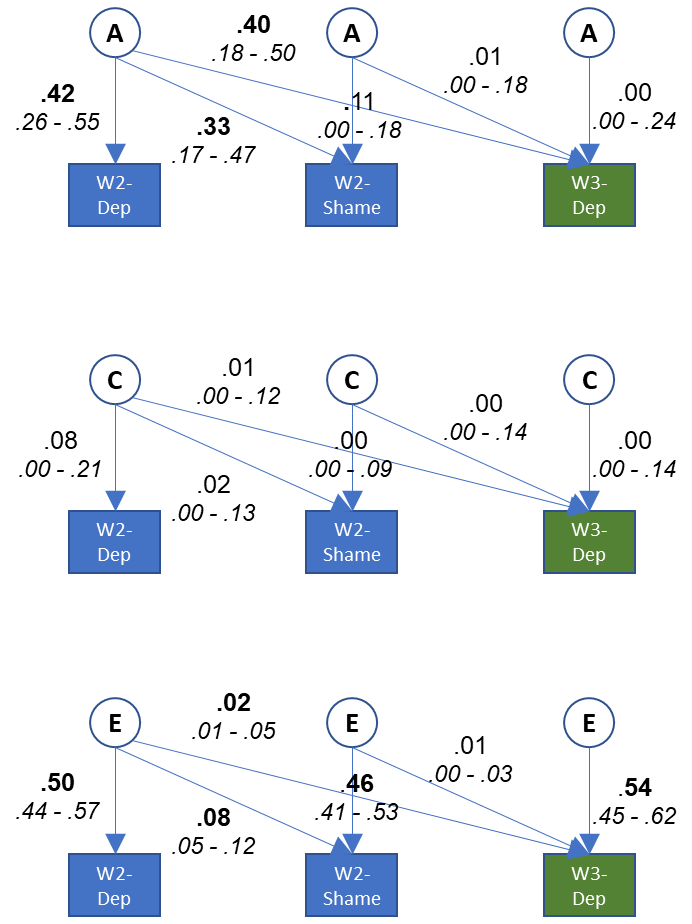
**

*Figure S1.* Results from Cholesky decomposition of prospective associations between shame and later depression, accounting for earlier depression. Note. A genetic effects, C shared environmental effects, E non-shared environmental effects. Significant values in bold face.
